# Striking Multiple Primary Tumors that underwent Whipple Procedure due to Periampullary Carcinoma: An Analysis of 21 Cases

**DOI:** 10.5005/jp-journals-10018-1249

**Published:** 2018-05-01

**Authors:** Osman N Dilek, Oguzhan Ozsay, Serkan Karaisli, Emine Ö Gür, Ahmet Er, Selda G Haciyanli, Haldun Kar, Fatma H Dilek

**Affiliations:** 1 Department of Surgery, Izmir Katip Çelebi University School of Medicine, Izmir, Turkey; 2 Department of Pathology, Izmir Katip Çelebi University, Ataturk Research and Education Hospital, Izmir, Turkey

**Keywords:** Diagnosis, Multiple primary tumors, Pancreas, Treatment, Whipple procedure.

## Abstract

**Introduction:**

The term multiple primary tumor (MPT) is used to describe cases where two or more primary tumors show no histopathological similarities in between. Multiple primary tumor cases have begun to increase in recent years as a result of the increase in life expectancy because of the increase in life standards and progress in diagnostic methods. In this study, MPT cases with periampullary tumors that underwent Whipple procedure were discussed in the light of literature data.

**Materials and methods:**

The patient files of 223 cases with periampullary tumors that underwent Whipple procedure in our hospital during the last 6 years were examined retrospectively. More than one primary tumor was detected in 21 patients.

**Results:**

Periampullary carcinomas were detected as a second primary tumor in 18 patients. First primary tumor was periampullary carcinoma in 3 patients that underwent Whipple procedure. After the Whipple procedure, 5 patients died due to early complications in the first 30 days and 6 patients died due to metastases and additional problems that developed during follow-up.

**Discussion:**

The incidence of MPT has been reported as 0.7 to 14.5% in the literature. Most of them are multiple primary case presentations. In patient management, it is recommended that each tumor should be evaluated independently of its own characteristics, and treatment and follow-up should be planned accordingly.

**Conclusion:**

The MPT cases are increasing. The possibility of MPT as well as metastasis should be kept in mind during the evaluation of tumor foci seen during diagnosis and follow-up of patients. The characteristics of each tumor, survival, and prognosis should be evaluated separately and the most appropriate treatment should be offered to the patient. It is recommended that synchronic primary tumors which are considered to be surgically resectable without metastasis should be removed in the same session.

**How to cite this article:** Dilek ON, Ozsay O, Karaisli S, Gür EÖ, Er A, Haciyanli SG, Kar H, Dilek FH. Striking Multiple Primary Tumors that underwent Whipple Procedure due to Periampullary Carcinoma: An Analysis of 21 Cases. Euroasian J Hepato-Gastroenterol 2018;8(1):1-5.

## INTRODUCTION

The term MPT is used to describe cases where two or more primary tumors show no histopathological similarities in between.

Multiple primary tumor cases have begun to increase in recent years as a result of the increase in life expectancy because of the increase in life standards and progress in diagnostic methods. In the United States, the number of cancer patients who are alive increased by more than three times since the 1970s. Since the first clinical trial of MPTs in 1934 by Bugher, more cases and publications on MPT have begun to appear in recent years.^1-3^

Multiple primary tumors can be synchronous or metachronous depending on the duration. Many environmental, genetic, and iatrogenic risk factors are thought to be involved in the development of MPT. It is proposed to question the criteria set by Warren and Gates to define MPT cases.^4,5^ According to this:

- Each lesion has to be malignant.
- It should be established that the pathological features of each lesion are different.
- The possibility of metastasis of the first lesion should be ruled out.

Multiple primary tumors can be seen in four different types according to their origin. These can be defined as multicentric MPTs arising from the same organ or tissue, systemic MPTs occurring in different organs and systems, MPTs that develop in double organs, and randomized incidental MPTs.^2,6,7^

The presence of other primary tumors involving other organs in patients with periampullary tumors and the clinical behavior to be followed in these cases should be investigated. Herein, we aimed to reveal the MPT cases and their clinical features between our patients who have undergone Whipple procedure due to periampullary tumor in our clinic during the last 6 years.

## MATERIALS AND METHODS

The records of 223 patients with periampullary tumors that have undergone Whipple procedure in our hospital in the last 6 years were retrospectively reviewed. Multiple primary tumor was detected in 21 of these patients (Table 1). The patients with second primary tumors who could not be operated due to the progression of tumors or accompanying comorbidities were not included in the list.

## RESULTS

Multiple primary tumor was detected in 21 patients. One patient with familial adenomatous polyposis (FAP) syndrome had five primary malignant tumors. About 20 patients had malignant tumors in different areas. The distribution of these malignant tumors is as follows: 8 colon/rectum tumor, 2 breast, 2 prostate, 2 stomach (one has gastrointestinal stromal tumor— GIST), 1 lung and bladder, 1 tumor with neural origin, lung, bladder, renal cell, pharynx melanoma and larynx tumor (Table 1). The mean age was 65.8 years (31-86). There were 4 synchronous and 17 metachronous MPTs among our cases. In three of our cases, two colonic and one laryngeal carcinoma were detected during the follow-ups after the Whipple procedure and they were operated. In all the other cases, Whipple procedure was performed due to the second primary malignant tumor originating from the periampullary region. The duration between the first primary tumor and the second primary tumor development varies between 0 and 22 years (mean 5.4 years). Five of the cases were lost due to complications during the first 30 days after surgery. Six of our cases were lost due to metastases and other reasons during follow-up. Ten of our patients are still alive.

## DISCUSSION

Those who have a tumor have a better chance of getting a second primary tumor than the normal population. The incidence of MPTs was reported as 0.7 to 14.5%. In the literature, there are many case reports with 2 to 7 primaries.^8,9^ There are publications reporting MPT cases as 18.4 to 33% synchronous and 66 to 81.6% as metachronous.^3,9,10^ Synchronous lesions were found in four of the patients in our series and this was less compared with the literature (Table 1).

The duration between MPTs varies widely, and there are data in the literature that vary between 0.5 and 28 years (mean 8.1 ± 2.5 years).^2,3^ In patients who receive radiotherapy and chemotherapy, this time is shorter and reported to be 3.2 + 3.7 years.^2,3^ In our series, this period varies from 0 to 22 years, with an average of 5.4 years.

Although there is no definite etiologic cause for MPT development, it is known that risk factors, such as increased genetic susceptibility, environmental factors, and iatrogenic risk factors, such as chemotherapeutic agents, hormonal agents, and radiotherapy that are used for diagnostic and therapeutic purposes play a role. Depending on the lifestyle, demographic factors, such as smoking, alcohol, nutrition, inactivity, and obesity also play a role.^2^

The role of cigarette in the development of pancreatic cancer is already known. The possibility of MPTs should always be considered in other cancer-related patients with cigarette smoking.^11,12^

It is known that genetic transition plays a role in 15% of pancreatic cancer cases. Multiple tumors can be seen in Gardner and Li Fraumeni syndromes that show genetic transition characteristics. Multiple primary tumor was reported in 21.5% of patients with Lynch syndrome.^13^ Punctiform mutation, loss of heterozygosity, and increased genetic instability may result in a lot of damage in several genes as a result of increasing environmental interactions in the technology age. Especially the mutations in TP53 gene also known as the tumor suppressor gene are also thought to play a role in the development of MPT. In a genetic case study by Romaniuk et al,^9^ they have demonstrated that cell proliferation potential and antiapoptotic stability are high in cases with MPT. There is no such genetic background study in our series. However, this may be the case for patients in whom we have had to perform Whipple procedure due to periampullary tumors.

The BEIR 7 model study of the American Health Council reported the lifetime attributable risk for patients treated with radiotherapy as 3/1.000, although it varied depending on the organs. This risk varies with age. It has been reported that in the younger patients who received radiotherapy due to malignancy, the risk of developing MPT may increase 4 to 60 times depending on the organs, compared with those who receive radiotherapy in advanced ages. Advanced age presents as an important risk factor.^14^

**Table 1:** **Table 1:** Demographic features of our patients with MPTs

*Age*		*Male/female*		*1st primary tumor*		*Procedures*		*2nd primary tumor*		*Definitive Procedures*		*PO status*		*Follow-up*	
72		Female		Left colon		Left colectomy, 8.2008 + CT		Main bile duct (distal)		Whipple 1.2010		Bleeding		PO excitus 1.2010
60		Male		Pancreas		Whipple 9.2010 + CT		Larynx		Laryngectomy 9.2011		Discharged		Live	
75		Female		Breast		Operation 4.2006 + CT		Pancreas		Whipple 6.2012		Fistulae		PO excitus 6.2012
66		Male		Pancreas		Whipple 9.2015 + CT		Left colon		Left colectomy 6.2017		Discharged		Live	
57		Male		Stomach		Synchronous tumors		Pancreas (Metastatic?)		Gastrectomy + Whipple 3.2015 + CRT		Discharged		Live	
55		Male		Right colon		Right colectomy 5.2009 + CT		Pancreas (Metastatic?)		Whipple 12.2010		Fistulae, sepsis		PO excitus 12.2010	
67		Female		Breast		Operation 1991 + CT		Pancreas		Whipple 12.2010		Discharged		Excitus 4.2012 Metastasis	
48		Female		Stomach (GIST)		Synchronous Tumors?		Pancreas Tm (Metastatic?)		Gastrectomy + Whipple 11.2012 + CRT		Discharged		Excitus 5.2016 (Pneumonia)	
66		Female		Sigmoid		Synchronous tumors		Ampulla tumor		Left colectomy Whipple 2.2012 + CT		Discharged		Excitus 4.2014 (Metastasis)	
72		Male		Ampulla		Whipple 6.2010 + CT		Rectosigmoid		Left colectomy, 3.2011		Discharged		Excitus 3.2011 (Metastasis)	
73		Male		Bladder + lung		Operation Bladder 2005 Lobectomy 3.2007 + CRTs		Pancreas		Whipple 9.2015		Discharged		Excitus 7.2017 (COLD)
64		Male		Lung		Operation, 2012 + CRT		Ampulla		Whipple 4.2015		Discharged		Live	
69		Male		Farinks Melanoma		Operation, 2008		Pancreas (IPMN)		Whipple 10.2015		Discharged		Live	
59		Male		Bladder		Operation, 2013 + CRT		Main bile duct (Distal)		Whipple 10.2015		Discharged		Live	
70		Male		Right colon		Synchronous tumors		Ampulla		Whipple + Right colectomy 11.2015 + CT		Hemodynamic instability? AMI?		PO excitus-11.2015
76		Male		Prostate		Operation, 2012 + CT		Klatskin (Type I)		Whipple 11.2015		Discharged		Excitus 10.2016 (Metastasis)
49		Female		Colon (FAP) Breast, Adrenal, desmoid		Total colectomy 2006, 2012, 2013, 2015, 2016		Pancreas Solid Pseudopapillary		Whipple 3.2016		Discharged		Live	
31		Male		Colon (FAP)		Total colectomy 9.2014		Duodenum		Whipple 12.2016		Discharged		Live	
79		Male		Prostate		Operation, 2009 + CRT		Ampulla		Whipple 11.2016		Discharged		Live	
86		Female		Schwannoma		Operation, 2010		Ampulla		Whipple 7.2016		Sepsis, MODS		PO excitus 7.2016
74		Male		Renal cell		Nephrectomy 1.2014		Pancreas		Whipple 7.2016		Discharged		Live	

PO: Postoperative; FAP: Familial adenomatous poliposis coli; CT: Computed tomography; CRT: Chemoradiotherapy; COLD: Chronic obstructive lung disease; MODS: Multiple organ dysfunction syndrome; AMI: Acute myocardial infarction; IPMN: Intraductal papillary mucinous neoplasms.

It is stated that the secondary primary tumors may develop iatrogenically after the chemotherapy, hormonal treatments, and radiotherapy for the treatment of the first primary tumor. Certain chemotherapeutics, especially alkylating agents, can cause impairments on deoxyribonucleic acid synthesis and functions.^15^ It is detected that in our series 12 patients received chemotherapy and 6 patients received radiotherapy, however, we do not have the data to show that it played a role in the development of secondary tumors.

Through advanced diagnostic tools, many previously unknown undifferentiated occult tumors can now be detected. In one of our cases, even though there was no clinical and radiological data, a second primary tumor was detected due to activity involvement in the pancreas during the positron emission tomography (PET) computed tomography (CT) scanning for primary colonic tumor. It has been reported that tumors may develop over the years depending on the rays taken during CT imaging, especially used for diagnostic purposes. In BEIR 7 studies in the USA, it was revealed that a patient who undergoes CT receives a mean of 10 to 20 mSv radiation and radiation causes cancer in one in every 600 patients who undergo abdominal CT.^14,16^ Computerized tomography taken during periodic postoperative follow-ups is likely to increase this risk.

It is emphasized that MPT develops especially after prostate, breast, colorectal, and bladder tumors.^2,3,17^ In our series, it is seen that 14 cases are in the group that is described in Table 1. The likelihood of having MPT in these cases is high and it may be because the incidence is higher in the community, the survival time is longer, and it may be due to periodic screenings. In a series by Cheng et al,^18^ colon, stomach, and liver lesions were reported as the most common tumors in the 129 MPT gastrointestinal stromal cases. In the same series, one-third of the same cases were found to be associated with gynecologic primary tumors and the number of cases of periampullary MPT was reported as 6. In the series of Irimie et al, the coexistence of breast-gynecologic carcinoma and colon-breast carcinoma was the most common case. Different demographics of countries may play a role in different coexistences.

Although there is a large number of case reports of MPTs involving colorectal carcinomas in the literature, we did not find any series with periampullary tumors with MPTs.^5,18^ In the US SEER data of 4,013 cases published by Amin et al^11^ from New York Presbyterian Hospital, they have found that the relative risk of pancreatic carcinoma is more in patients aged 20 to 49 years with colon or hepatobiliary tumors, in patients with pharynx, colon, or stomach tumors aged 50 to 64 years or in patients with biliary or gastric tumors aged 65 years and above. The patients who were referred to our clinic to undergo the Whipple procedure were usually the second primaries. In our series, colorectal tumors were the most common type of MPTs seen with periampullary tumors (eight cases). In our series, colon and laryngeal carcinomas were detected only in three cases during follow-ups after Whipple surgery. This might be because of the short duration of survival with periampullary tumors. Neugut et al^19^ and Neugut and Gold^20^ reported that lung and head and neck cancers may be more common, particularly in cases of pancreatic cancer who are smokers. Laryngeal carcinoma was detected 1 year later in one of our patients who underwent Whipple procedure who was a smoker and he was operated.

It has been reported that PET CT may be helpful in the detection of metastases and in the differential diagnosis and detection of secondary primary tumors.^21^ In two of our patients with lung cancer and one patient with laryngeal cancer who were smokers, primary pancreatic tumor was detected during follow-ups and they were operated. The PET CTs taken during the follow-ups of these patients have played a role in the diagnosis of the second primary tumor. In one of our cases, pancreatic involvement was detected with a control PET CT when there was no pancreatic mass image. After biopsy and Whipple procedure, the tumor was diagnosed as a solid pseudopapillary tumor.

It is recommended to follow the oncological surgical principles in the treatment. Each case should be evaluated according to the performance status and tumor stage. As with the first tumor in MPT cases, it is recommended to plan and implement treatment interventions at the earliest possible stage in secondary lesions.^3^ Patients with malignancy are recommended to follow a specific algorithm in terms of local recurrence and metastasis.

As a result, the possibility of MPT during the evaluation of patients in the preoperative period should be kept in mind. The risk of developing MPT is greater in younger patients with primary tumors. The longer the lifespan, the greater the risk of developing MPT. The coexistence of smoking-related tumors in smokers should be considered first. Early diagnosis is seen as the most important factor that affects survival. The treatment, survival, and prognosis of each tumor should be evaluated separately. However, in the treatment of the second primary tumor, the prognosis of the first primary tumor and the performance status of the patient are the most important determinants. It is recommended that surgical interventions in synchronous cases should be performed in one session, if possible.
